# Biodiversity Effects on Human Mental Health via Microbiota Alterations

**DOI:** 10.3390/ijerph191911882

**Published:** 2022-09-20

**Authors:** Yee Sang Wong, Nicholas John Osborne

**Affiliations:** 1 School of Medicine, The University of Queensland, Herston, QLD 4006, Australia; 2 School of Public Health, The University of Queensland, Herston, QLD 4006, Australia; 3 School of Population Health, University of New South Wales, Sydney, NSW 2052, Australia; 4 European Centre for Environment and Human Health (ECEHH), University of Exeter Medical School, Knowledge Spa, Royal Cornwall Hospital, Truro TR1 3HD, Cornwall, UK

**Keywords:** ecosystem biodiversity, neuropsychiatric disorders, gut microbiome

## Abstract

The biodiversity hypothesis postulates that the natural environment positively affects human physical and mental health. We evaluate the latest evidence and propose new tools to examine the halobiont environment. We chose to target our review at neuropsychiatric disorders, including depression, anxiety, autism, dementia, multiple sclerosis, etc. because a green prescription (exposure to green spaces) was shown to benefit patients with neuropsychiatric disorders. Specifically, our review consists of three mini reviews on the associations exploring: (1) ecological biodiversity and human microbiota; (2) human microbiota and neuropsychiatric disorders; (3) ecological biodiversity and neuropsychiatric disorders. We conclude that the environment could directly transfer microbes to humans and that human studies support the gut microbiota as part of the pathophysiology of neuropsychiatric disorders. Overall, the results from the three mini reviews consistently support the biodiversity hypothesis. These findings demonstrated the plausibility of biodiversity exerting mental health effects through biophysiological mechanisms instead of psychological mechanisms alone. The idea can be further tested with novel biodiversity measurements and research on the effects of a green prescription.

## 1. Introduction

Neuropsychiatric disorders, mental illnesses caused by organic brain diseases, affect almost one billion individuals globally [1]. They include multiple sclerosis, autism spectrum disorder, anxiety, depressive disorders, and more. Ongoing costs to the global economy are estimated at AUD 2.5 trillion annually, which are expected to rise [1]. Faced with significant barriers to the accessible provision of available treatments, a key focus will be to search for scalable, approachable, and inexpensive interventions.

**Figure 1:**
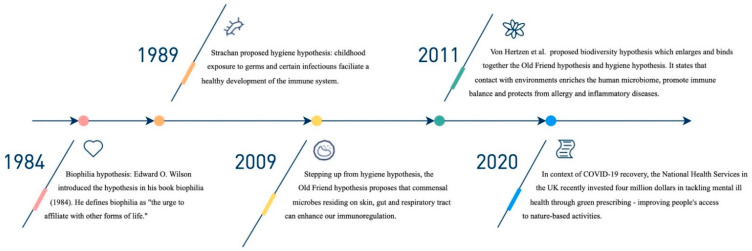
Key events important to the biodiversity hypothesis.

Utilising exposure to microorganisms as potential sources of prevention has been an ongoing endeavour with recent notable advancements (see Figure 1). Originating in 1989, Strachan’s hygiene hypothesis attempted to explain why immune-mediated diseases were on the rise in nations with lower infection rates [2,3]. According to Strachan, the prevalence of atopic disease was related to an increased number of siblings [2]. Thereafter in 2009, Rook proposed the Old Friends mechanism behind the hygiene hypothesis [4]. To summarise similar theories that followed, von Hertzen et al. proposed an amalgamation of microbiome-based hypotheses termed the “biodiversity hypothesis” [5]. According to Rook, the co-evolution of commensal organisms is the basis for the mechanism of biodiversity theory [4]. Co-evolution provides immunoregulatory training by stimulating T-regulatory cells that turn off an inappropriate attack on self [4]. For instance, it appears that helminths can enhance immunoregulation. When patients with multiple sclerosis are infected by helminths, their disease stops progressing, an exciting finding that has led to recent clinical trials [6]. A research frontier in neuropsychiatric illnesses is to ameliorate or prevent inappropriate immune/inflammatory responses.

The 2007 launch of the Human Microbiome Project (HMP) marked a turning point for recent significant advancements in studies of the human microbiota [7]. The HMP investigations characterised the microbial communities that live within people [7]. It fuelled research into the mechanisms of the gut–brain axis, a previously undiscovered bidirectional link between the brain and the gut [8]. Our gut flora is essential to how we live and how the brain works. Parallel research projects on the microbiota of mentally disordered patients were made possible by the HMP [9]. The HMP also sped up the development of genetic sequencing technologies (omics), which will continuously produce more data for further studies.

Our paper examines whether environmental biodiversity can affect our mental health by altering our microbiota. In our review, we dissect the research question into three mini-reviews. Each mini-review focuses on an association between (1) ecological biodiversity and human microbiota; (2) human microbiota and neuropsychiatric disorders; and (3) ecological biodiversity and neuropsychiatric disorders. We decided to focus our review on neuropsychiatric illnesses, which are mental and neurological conditions with biological and physiological roots. To the best of our knowledge, studies to date have focused on psychological mechanisms underlying biodiversity health effects while our paper focuses on physiological mechanisms. We also chose to target the human microbiota because there is little inheritability (1.9%), primarily affected by the environment, and thus potentially modifiable [10]. Other authors have identified the following factors that influence microbiota composition: gender [11], immunodeficiency [11], hyper-immunity [11], alcohol [11], diets [11,12], genetics [11,12], breastfeeding [12], mode of birth [12], medications [11,12], and animal ownership [13].

## 2. Materials and Methods

In March 2022, we identified studies listed in PubMed and the Web of Science, common databases for epidemiological and human health studies databases. We identified keywords that focused on the three associations of interest: (1) ecological biodiversity and human microbiota; (2) human microbiota and mental health; (3) ecological biodiversity and mental health. We created three search strings using combinations of pairs from the following three sets of key terms: set a: ‘biodivers*’ or ‘species richness’; set b: ‘microbiota’ ‘microflora’ or ‘*-brain axis’ or ‘skin flora’; set c: ‘mental health’, or ‘psychiatric’ or ‘well-being’ or ‘anxiety’ or ‘depression’ or ‘dementia’ or ‘schizophrenia’. The terms ‘human’, ‘transfer (of microbes)’ or ‘environment* (microbes)’ were added to improve accuracy. These sets were searched in pairs—a AND b, b AND c, c AND a. These searches were conducted with no restrictions on the year of publication. The title and abstract of each potentially relevant article were screened. Papers were considered for detailed evaluation if the neuropsychiatric disorders were the primary focus of the study, the subjects studied are human, they were written in English, and excluded if they were studies involving comorbidities.

The combined three searches initially yielded 349 papers. After screening the abstract, most of these were excluded because they were unrelated to the research topic. Many studies and reviews focused on involvement of microbes in ecosystem services (i.e., wastewater management, resources, plant diseases, wildlife support) or mental health as represented by the feeling of well-being with no diagnosis. A total of 18 studies met inclusion criteria. Seven papers that studied environmental biodiversity and human microbiota published between 2012 and 2021 met the inclusion criteria. Three articles published between 2015 and 2020 that reported environmental biodiversity and mental health met the inclusion criteria. Eight reviews published between 2012 and 2021 studied human microbiota and neuropsychiatric disorders, thus meeting the inclusion criteria. For this last set (set c), only reviews, systematic reviews and meta-analyses were considered given the breadth of the research in molecular biology. Additional papers were extracted from references of these papers.

## 3. Results

From the associated studies, we classified the evidence into three categories by their studied associations: the effect of ecological diversity on neuropsychiatric disorders, the mechanisms through which environmental plant diversity affects human microbiota, and the mechanistic link between the human microbiota and neuropsychiatric disorders. The separation of categories allowed us to provide a detailed examination of each of the three components of the theory.

### 3.1. Epidemiological Evidence of Ecological Biodiversity Effect on Neuropsychiatric Disorders

#### 3.1.1. Areas of Agreement

There were few epidemiological studies on the link between biodiversity and neuropsychiatric disorders. Of three studies returned in the search [13,14,15], two agreed that environmental biodiversity was associated with resilience against neuropsychiatric disorders [14,15]. Methorst et al., who conducted a sizeable cross-sectional analysis (population = 13,328) in all counties of Germany, found a positive relationship between plant and bird richness and a decrease in population-level depression and anxiety disorders [15]. Based on their calculations, a 10% increase in plant species richness (mean of 1134 plant species per county of mean area size = 973.9 km^2^) is related to an increase in the Mental Component Survey (MCS) score of 3.27 (CI: 1.56 to 4.99) from a mean score of 50.83. The MCS measures anxiety, depression and anxiety disorder prevalence in the general population, where a score equal to or below 45 is 83% specific and 87% sensitive to depression, and a score equal to or below 50 is 73% specific and 81% sensitive to anxiety disorders [16]. Similar effects on MCS score were measured for a 10% increase in bird species richness from a mean of 104.8 bird species per county. Methorst et al. accounted for various confounding factors, including the big five: personality, income and the ‘luxury effect’ (income, education level, and employment status). This is important because access to abundant green space is associated with higher living standards [15].

Bobel et al., in a small case-control study, found that urban dwellers without pets had more prolonged and elevated inflammatory responses compared to rural dwellers living with farm animals when subjected to a standardised psychosocial stress test, the Trier social stress test (TSST), measured by serum IL-6 and peripheral blood mononuclear cell (PBMC) counts (sample size was 20 for each group) [13]. PBMC are various specialised immune cells, specifically lymphocytes, dendritic cells and monocytes, that increase in number in an immune response. While the rural participants had a transient immune activation for PBMC count at 5 min after the stress test, urban participants had elevated PBMC count for 120 min, until the end of the study. A similar result was found for IL-6 levels, where the urban participants’ IL-6 levels remained high up to 120 min, compared to 90 min for rural participants. These participants were otherwise similar—no diagnosable neuropsychiatric disorders (as determined by DSM-IV and other physical chronic condition interviews), within the 20–30 BMI range, similar IL-6 levels and PBMC counts at baseline, and free of developmental or recent trauma. It is known that people with major depressive disorder and early life stress have elevated IL-6 levels in response to TSST. This study strengthens the idea that the environment is partially responsible for immune regulation. However, the veracity of this study may be confounded by sampling/random error due to its limited sample size.

#### 3.1.2. Areas of Controversy

By contrast, Marselle et al. found that tree density rather than tree species richness was correlated with antidepressant prescription (sample size = 9751) [14]. Tree density within 100 m of domicile, but not any further, reduced antidepressant prescription in people of lower socioeconomic status (SES) with a log OR of −0.21 (standard error 0.08 and *p* = 0.01). Marselle et al. also observed that low SES groups who live near areas of dense tree growth have similar rates of prescription antidepressants as those in the high SES group [14]. The luxury effect was excluded as there was a higher tree density near the low SES group in this sample. The study was well-designed as it controlled for protective factors including employment, age and optimism, and risk factors for depression such as spring, winter, tobacco smoking, being female, overweight or obese. The authors proposed that the result could be explained by psychological restoration from viewing nearby trees since trees have the effect of bringing higher attentional functioning in low SES groups as determined from previous studies. This study, being cross-sectional, has a self-selection bias. Healthier people may choose to live in areas with more green space that facilitate their healthy lifestyle, especially if they have high disposable incomes [17].

#### 3.1.3. New Research Frontiers

Marselle et al. and Methorst et al. attributed the positive effects to psychological mechanisms of positive emotions and attention restoration [14,15], while Bobel et al. attributed the positive effect to microbiome regulation by the environment [13]. Yet, all studies raised the difficulty of studying the effects of these mechanisms independently. Future studies could use a novel epidemiological approach by subtracting one effect from the overall association [18], for example, measuring the biological health benefit of biodiversity by removing the psychological benefit of biodiversity and other benefits of ecological services from the overall benefit observed, or vice versa.

It remains debatable whether diversity or quantity of greenspace reduces the prevalence of neuropsychiatric disorders. Marselle et al. proposed quantity may matter more than biodiversity in locations that are already sparse in vegetation, therefore, the quantity may have more positive effects in areas that have low vegetation overall [14]. Inconsistencies may also be due to differences in data collection between morning versus afternoon [15]. Data on bird species recorded in the afternoon tended to be richer but species data are usually collected over years at different times of the day [19]. None of the studies above measured microbiota diversity directly, and it is not clear how well plant, bird or other animal diversity represents exposure to microbiota diversity exposure.

To examine the biodiversity hypothesis for these studies, a few assumptions need critical evaluation: (1) rural areas are more biodiverse and built-up areas are less so, and (2) richness of plant and bird species richness is an indicator of overall landscape biodiversity. Although the rural–urban dichotomy for biodiversity is intuitive, limited literature has confirmed a more diverse microbial composition for rural environments than urban centres. If the urban environment is indeed less biodiverse, it would be interesting to examine the peculiar dominance of certain neuropsychiatric disorders in urban areas, such as schizophrenia, in the context of the biodiversity hypothesis [20,21].

Lastly, there is no gold standard for the representation of biodiversity. Inclusive reporting of abundance, richness, evenness, and diversity is needed to prevent biases. Inconsistency of data and variable exposure and case definitions leave room for linkage flexibility and results in the dangers of associating these with health outcomes. Studies cited in this review show that sometimes effects are related to the richness, but not diversity [22], or sometimes both [23]. However, not all studies report both. Researchers may also explore alternative known methods to estimate microbial and plant diversity where diversity information is unavailable. For example, pH is an accurate predictor of soil microbial diversity [24]. A specific type of fungi diversity (mycorrhizal) is associated with plant diversity and productivity as they are crucial for plant growth and survival [25].

### 3.2. Environmental Microbes and Human Microbiota

#### 3.2.1. Areas of Agreement

Seven studies [10,22,23,26,27,28] found that human microbiota is influenced by environmental biodiversity. Two studies [22,23] observed the transfer of environmental microbes to nasal and skin microbiota. Selway et al. swabbed skin and nasal microbiota before and after subjects interacted with urban green space for 15 min and found that nasal and skin microbiota were more diverse after exposure [23]. Skin microbiota were more similar to the soil microbes, while nasal microbiota were more similar to air samples. The research documented microbial mobility between the environment and human microbiota after exposure. In another study, Lai et al. attributed 3.1 ± 1.9% of the nasal microbiome and 3.0 ± 1.5% of the skin microbiome to the working environments of animal workers [22]. However, these research sample sizes were small, with three and ten subjects, respectively.

Sun et al. and Jin et al. found that gut microbiota changes in humans after exposure can last for months [27,29]. Sun et al. found that the gut microbiota of veterinary students who lived on a farm for three months took four to six months to return to their original composition after they left the farm [29]. In a study by Jin et al., two out of five expeditioners to Antarctica maintained a similar intestinal microbiota to what they developed in Antarctica for at least one month after returning to their home country [27]. We already know that environmental factors, especially diet, change the microbiota composition, but neither of these studies excluded dietary effects [30]. Further research on exposure effects should preferentially adopt observation of diet after leaving the exposure site to exclude its possible effect after exposure.

Hanski et al. and Pearson et al. [26,28] found an association between the surrounding land types and the diversity of the human microbiota. Hanski et al. [26] found that residents near forests and farmlands but not near buildings and water bodies have higher skin microbial diversity. Pearson et al. [28] found that living on loam soil types was associated with increased human intestinal microbial diversity compared to clay soil types, but the microbial diversity of clays was not measured. Land type studies are promising areas of research as they utilise freely available large-scale data and elucidate the assumed positive correlation between rural land types such as forests, and more diverse human microbiota.

Two authors [10,27] found that individuals living in a shared space develop similar intestinal microbiota. Rothschild et al. found that unrelated individuals living in the same household develop a similar diversity and species of microbiota, albeit maintaining a level of individuality. Rothschild et al. further quantified this using a United Kingdom twin study, showing that the heritability of microbiota was 1.9% in a study of 2252 twins, whilst the rest of the microbiota were attributed to other factors, including the environment [10]. Jin et al. found that six expeditioners to Antarctica developed some similarities in microbiota after they reached Antarctica together within a month [27]. These data together begin to challenge the concept of stability of gut microbiome composition over a lifetime from development in the first few years of life and adds evidence of the effect of migration on the microbiota.

#### 3.2.2. Areas of Controversy

The human gut microbiome is considered relatively stable over a lifetime once established [30]. The challenges lie in discovering the most dynamic confounders and controlling for them in future studies. This may be the reason that studies disagree over what confounders to control. These confounders may include, antibiotic use, diet, and temperature [11,27]. As the microbiome becomes less expensive and quicker to sequence, data interactions between the environment and human microbiota will become more available offering a potentially greater understanding of their mechanisms.

Studies also disagree as to whether it is diversity or specific species that might benefit human microbiota. It cannot be ignored that it may be the particular species of microorganisms, rather than their diversity that benefit the human microbiota. This is a work in progress as the ecology of the gut/skin microbiota is extremely complex. Firstly, the scale required for human microbiota studies is immense. There are about 3.3 million non-redundant genes in the gut microbiome alone, as compared to 22,000 genes in the entire human genome [30]. Secondly, is diversity or individual species of microbiota more important? There is insufficient data to conclusively answer this. Different microbial species may yield similar functional physiology. Additionally, studies suggest that the ratio of bacterial species characterises the status of the obese gut microbiome rather than diversity [30]. Thirdly, there is increasing data to suggest that healthy core microbiomes exist. However, for most people, human skin and gut microbiomes are 80–90% different from one another [30]. What environmental microbiomes there are for an individual may vary highly and this is emphasised by humans living healthily in very different environments (city, desert, jungle, ice) and each with a different microbiome [30].

#### 3.2.3. New Research Frontiers

The gap in research includes studies of behavioural interactions allowing humans to acquire these potentially beneficial microbes from the environment. Activities must be documented, including touching the nose, and eating or drinking in the environment of exposure. The other gap in research is the exact path microbes take to travel from the environment to the human gut. Air microbiota can affect the diversity of gut microbiota in mice [31], but no human studies to date have traced bacterial pathways. It was proposed that, in humans, mucociliary escalators sweep debris, including microorganisms, into our oral pharynx and this later gets swallowed into the gastrointestinal tract [32].

### 3.3. Microbiota in Neuropsychiatric Disorders

Microbes are everywhere in the human body, including the nose, skin, gut, and what were previously thought to be sterile lungs [33]. The hypothesis is that a healthy individual has a diverse commensal microbiota at these sites that support their homeostasis. In contrast, dysbiosis, the perturbations of such commensal microbial communities, causes functional disturbance and diseases [11].

#### 3.3.1. Areas of Agreement

All eight reviews [8,34,35,36,37,38,39,40] ranging from 2016 to 2021 agree that the links are apparent between gut microbiota and neuropsychiatric disorders and are confirmed in animal models, although no causal inferences were made. Animal studies demonstrated possible pathophysiological pathways in autism, anxiety, major depressive disorders, multiple sclerosis, Parkinson’s disease/Alzheimer’s disease, schizophrenia, bipolar disorders, delirium, migraine, and anorexia nervosa (Table 1). For those with autism and bipolar disorder, gut microbiomes differ in composition from those of healthy individuals. They may correlate with the severity of symptoms [38]. Dysbiosis is observed as anxiety-like and depression-like behavioural changes in both human and animal models [38,40]. These findings substantiate the microbiota–organ component of the biodiversity hypothesis. No studies on the link between either the lung–brain axis or the skin–brain axis linked with neuropsychiatric disorders were found.

The authors also agreed that animal studies revealed possible mechanistic pathways underlying the microbiota–brain interactions. These mechanisms include:

1. Direct activation of the vagus nerve [8];
2. Derived nutrients reaching the brain [8,34];
3. Maintenance of brain cells (microglia) [34];
4. Participation in immune regulation and release of inflammatory mediators [8,34];
5. Disruption of maternal microbiome altering foetal neurodevelopment [39].

These discoveries support the theory that the gut–brain axis and its regulation by the microbiota may play a vital role in the biological and physiological basis of neuropsychiatric disorders. This provides evidence for more mechanisms than what Rook proposed for the Old Friends hypothesis, in which he focused only on immunoregulatory mechanisms. 

The authors agreed that the hypothesis of microbiota playing a pivotal role in neuropsychiatric disorders was plausible in the development of neuropsychiatric diseases. For example, more than 50% of the neurobiological factors involved in autism are driven by non-inheritable causes [35]. Oxytocin improves social behaviour with specific probiotic bacteria increasing the pituitary production of oxytocin [35]. In anxiety and depression, we know that dysregulation of neurotransmitters underlies the pathophysiology of the disease, and gut microbes produce a measurable neurotransmitter [34]. As another example, multiple sclerosis is an autoimmune disease that attacks the human nervous system and is characterised by chronic inflammation. The lack of individual species of gut microbiota reduces the capacity to attenuate immune cell activities [36]. In anorexia nervosa, dysbiosis of Escherichia coli, specifically in the gut microbiome, leads to the secretion of the ClpB protein, which increases the drive for slimness or interpersonal distrust [37].

#### 3.3.2. New Research Frontiers

As the microbiota–gut–brain axis is a two-way pathway, altered microbiota may be a manifestation rather than a cause of the illness. This makes research design challenging, and the only way to confirm a causal link may be through the use of interventional studies. Early human interventional trials with probiotics, antibiotics and faecal microbiota transplant (FMT) showed mixed results in ASD, MDD, bipolar, Alzheimer’s and Parkinson’s disease [38]. Challenges include identifying strains and the composition of microbiota that might be beneficial to humans.

There is still considerable debate as to whether the gut microbiota changes are core to such conditions or are merely epiphenomenal [35]. For most of the reviewed neuropsychiatric disorders, the corresponding gut microbiota differed from the control population, at least with animal studies. Animal studies also provided many possible mechanisms making this hypothesis plausible. It would be premature to interpret these studies in the context of environmental biodiversity. Still, interdisciplinary reference to this field of study is recommended to understand how ecological biodiversity could be interrelated.

The lung–brain axis may affect our interpretation of gut microbiota research emerging in the past few years [33]. Some associations were found between neurodegenerative conditions and lung inflammation. For instance, an increased risk of Parkinson’s disease (PD) and Alzheimer’s disease (AD), which are both associated with Chronic Obstructive Pulmonary Disease (COPD), are thought to be correlated with an altered lung microbiome and traffic pollution [41]. Lung–brain axis research is in its early stage. The lung was previously described as sterile. However, enabled by bronchioalveolar lavage, researchers discovered that the lungs host similar microbiota to the gut [33].

In summary, whereas evident associations exist for (1) ecological biodiversity and human microbiota and (2) human microbiota and mental health, there is relatively limited epidemiological evidence to support the associations between ecological biodiversity and mental health.

## 4. Discussion

In the first mini-review, three epidemiological studies showed that exposure to diverse plants and animals has a positive impact by reducing the risk of neuropsychiatric disorders [13,14,15]. From these studies, we conclude that nature can increase population resilience against neuropsychiatric illnesses but it is not clear whether this resilience is achieved through microbial transfer. However, an important limitation identified is whether plant and bird species richness is a suitable proxy measure for microbial diversity, as many of these proxy measures are not yet validated.

The second mini-review aimed to answer whether a biodiverse environment directly introduces environmental microbes to the human skin, nose and gut [10,22,23,26,27,28]. These studies confirm that the environment could directly transfer microbes to humans even during brief interactions.. These studies also demonstrated that short-term (minutes to hours) exposure studies tend to have better validity than long-term (months) exposure studies. This is due to our lack of knowledge and lack of ability to exclude confounding factors such as diet and antibiotic use. Long-term exposure studies were, however, able to show effects lasting months. This provides insight into the possible long-lasting nature of biodiversity health effects.

The third mini-review catalogued various physiological pathways in which the human gut microbiota and brain interact and participate in disease processes of neuropsychiatric disorders [8,34,35,36,37,38,39,40]. Although the studies listed in Figure 1 were limited by their small sample sizes, they jointly showed the potential benefits of microbiota-based interventions.

The key limitation of this review is the inclusion of review articles in mini-review three. The decision was made given the breadth of molecular biological studies on the topic of the gut–brain axis. While we believe these limitations have not impacted the primary outcome of the study, future work could seek to narrow the topic to only examine primary studies.

Future studies would be aided by the use of environmental DNA (eDNA) with metabarcoding technology for time-efficient and direct measurement of microbial diversity. eDNA is a non-destructive approach to sample DNA of known species found in the environment, whilst metabarcoding associates sequences from eDNA with a taxonomic name [42,43]. Water, air, and soil eDNA can predict the presence of insects, plants, and animals with higher sensitivity than visual evaluations [44]. Most importantly, eDNA addresses data validity concerns when using non-microbial species as a proxy for measuring microbial diversity. Fast and direct measurements also enable the collection of fine spatial data, such as height, which may yield significant findings given microbe diversity has previously been found to be greatest between 0.5 and 2 metres above ground [45].

Extending this into policy applications, the biodiversity hypothesis may explain the causal mechanisms behind the health benefits of a green prescription, where patients are prescribed outdoor activities with exposure to nature [5]. For example, in the context of post-COVID-19 recovery, the National Health Service in the UK recently invested 4 million pounds in tackling mental ill health through green prescribing—improving people’s access to nature-based activities such as local nature walks and community gardening [45]. While the health benefit of exercise is established, the green prescription is believed to have additional benefits including improved mood and decreased fatigue, and over-exercising indoor [46]. Therefore, our result provides additional support for urban green spaces, to not only target the psychological but also physiological mechanisms of species richness to improve public health outcomes.

## 5. Conclusions

The results from the three mini reviews consistently support the biodiversity hypothesis. These findings demonstrated the plausibility of biodiversity exerting mental health effects through biophysiological mechanisms instead of psychological mechanisms alone. They showed that the environment could directly transfer microbes to humans even during brief interactions in urban green spaces. Moreover, human interventional studies support connections between gut microbiota as part of the pathophysiology of neuropsychiatric disorders. Further studies into these additional mechanisms could hasten the development of biodiversity-based therapies as accessible and affordable treatment options for neuropsychiatric disorders.

## Figures and Tables

**Table 1:** Microbiota-based interventions and their effect on the various neuropsychiatric disorders.

		Microbiota Alterations		Interventional Outcomes
**Anxiety**			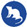 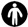	Symptoms ↓ with *Lactobacillus* and *Bifidobacteria* probiotic [8,37,38,40] Symptoms ↓ with *Lactobacillus*, *Bifidobacterium* and *Streptococcus* genera [38]
**Depression**	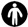 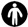	↑ Firmicutes and ↓ Bacteroidetes, Proteobacteria and Actinobacteria [35] ↓ *Bacteroides*, *Coprococcus* and *Dialister* genera [38]	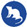 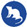 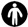 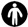	↓ Depression and anxiety symptoms with *Lactobacillus rhamnosus* and *Lactobacillus helveticus* [36] Symptoms ↓ with probiotic *Lactobacillus rhamnosus* [8,36] Symptoms ↓ with *Lactobacillus acidophilus* and *casei* and *Bifidobacterium bifidum* probiotics [34] Symptoms ↓ with prebiotics + probiotics [34]
**Bipolar**	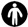	↓ *Faecalibacterium* and *Ruminococcaceae* genera [38]	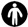 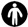	↓ Hospitalisation frequency after mania episodes with probiotics *Lactobacillus rhamnosus* strain GG and *Bifidobacterium animalis* subsp. *lactis*) [38] ↑ Incidence of mania with antibiotic use [38]
**Autism**	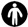 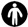 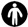 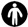	↑ Bacteroidetes/Firmicutes ratio [35] ↑ Microbial metabolites (SCFAs) and ↓ microbiota metabolite 5-aminovaleric acid [38] ↑ *Faecalibacterium*, *Lactobacillus*, *Bacteroides*, *Prevotella*, and *Alistipes* genera [36,40] ↓ *Prevotella*, *Bacteroides*, *Bifidobacterium*, and *Escherichia* genera, and *Akkermansia muciniphila* [36,38,40]	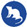 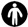 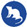 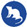 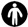	Symptoms ↓ with *Bacteroides fragilis* in mice [35,38,40] Symptoms ↓ Faecal Microbiota Transplantation [8,38] Symptoms ↓ with *Lactobacillus reuteri* [38] Symptoms ↓ with microbiota metabolite 5-aminovaleric acid [38] Symptoms ↓ with prebiotic Bimuno® galactooligosaccharide [38]
**Dementia and cognitive** **impairment**	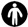 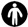 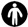 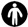 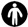 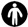	↓ Prevotellaceae in Parkinson’s disease (PD) [35,38,40] ↓ *Faecalibacterium prausnitzii*, *Lactobacillaceae*, *Enterococcaceae*, and Bacteroidetes Prevotellaceae; ↑ *Akkermansia muciniphila*, *Bifidobacterium* and *Enterobacteriaceae*; ↓ SCFAs such as acetate, propionate, and butyrate in PD [38] ↑ *lactobacillus* levels; ↓ *Clostridium coccoides* group, *Clostridium leptum* subgroup and *Bacteroides fragilis*; ↓ hydrogen-producing fecal bacteria in PD [38] ↓ butyrate-producing and anti-inflammatory bacterial genera such as *Blautia*, *Coprococcus*, and *Roseburia* in PD [40] ↑ *Oscillospira* and *Bacteroides*, *Ralstonia* in PD [40] ↑ *Helicobacter pylori* in Alzheimer’s disease (AD) [40]	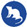 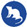 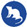 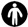 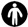 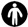	*Bifidobacterium longum* 1714 ↑ cognition [35] Antibiotics ↓ Aβ plaque pathology [38] Probiotic ↑ spatial memory in mice with Ab plaque [38] Three months of doxycycline and rifampin ↓Alzheimer’s disease severity [38] Probiotic ↑ cognitive function in Alzheimer’s disease (AD) [38,40] Symptoms ↓ with *Lactobacillus* and *Bifidobacterium* species via fermented milk in Alzheimer’s disease [40]
**Multiple** **sclerosis**	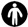 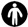 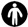	↑ *Escherichia*, *Shigella, Clostridium, Eubacterium rectal, Corynebacterium, Firmicutes, Psuedomonas, Haemophilus, Blautia, and Dorea* and ↓ *Parabacteroides, Adlercreutzia* and *Prevotella* genera [36,40] ↓ Metabolites (lipid 654) of Bacteroidetes [36] ↓ Firmicutes and Bacteroidetes [40]	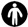 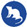	Probiotics ↓ inflammatory cells in patients [36] ↓ *Faecalibacterium* and *Fusobacterium* [36,40]
**Visceral pain**			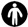 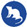 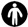 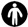	↓ pain with *Lactobacillus* [37,40] ↓ pain with *Bifidobacterium infantis* 35624 and *Lactobacillus farciminis* [40] ↓ pain with *Lactobacillus salivarius* UCC4331 [40] ↓ pain with *Bifidobacterium infantis* 35624 [40]
**Migraine**	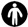	↑ *Helicobacter pylori* [40]		
**Anorexia** **nervosa**	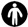 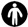	↓ *Roseburia* spp., ↑ *Methanobacterbrevi smithii*, Verrucomicrobia and *Bifidobacteria* [37] ↑ metabolites ClpB of *Escherichia coli* [37]		
**Schizophrenia**	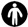	Gut microbiota was implicated in the reductive metabolism of psychotropic medications [39]	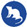 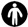	↓ Ampicillin [35] No differences were found in schizophrenia symptoms in the PANSS score between probiotic and placebo supplementation [38]
**Legend**	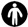 Observations made in human; 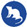 Observations made from animal studies; PD: Parkinson’s disease; AD: Alzheimer’s disease; SCFAs: Short-chain fatty acids			

## Data Availability

Not applicable.
